# Psychotherapy as investigation: cultivating curiosity and insight in the therapeutic process

**DOI:** 10.3389/fpsyg.2025.1603719

**Published:** 2025-07-23

**Authors:** Judson A. Brewer, Fabio Giommi

**Affiliations:** ^1^Department of Behavioral and Social Sciences, School of Public Health, Brown University, Providence, RI, United States; ^2^Department of Psychiatry, Warren Alpert Medical School at Brown University, Providence, RI, United States; ^3^Nous-School of Specialization (PsyD) in Psychotherapy, Milan, Italy; ^4^AIM-Italian Association for Mindfulness, Milan, Italy

**Keywords:** psychotherapy, curiosity, predictive processing, mindfulness, training, coaching, monitoring

## Abstract

Psychotherapy training and practice have grown increasingly complex, driven by expanding diagnostic frameworks, theoretical models, and intervention methods. This complexity can at best confuse and at worst overwhelm therapists, limiting therapeutic effectiveness and obscuring the relational core that is crucial to successful therapy. In this paper, we explore a different path than increasing complexity: simplicity. Specifically, drawing on recent insights from psychotherapy research and neuroscience, we highlight the intimate connection between simplicity and curiosity and how different types of curiosity can be operationalized in the therapeutic setting. We also explore how curiosity interacts with generative models or narratives that patients (and therapists) can be overidentified with, leading to confirmation bias. Also, we highlight how curiosity and simplicity can mutually foster each other in the therapeutic relationship, co-emerging as a strong driving factor for therapeutic insight and change. Further, we explore the relationship between curiosity and interoceptive awareness, which can be operationally enhanced by embodied practices (e.g., mindfulness, meditation etc.). Ultimately, rather than the accumulation of knowledge, psychotherapy that centers on curiosity empowers clients toward exploration and adaptive flexibility to foster autonomy and insight, while potentially protecting psychotherapists from stagnation, fostering continued personal and professional growth.

## Introduction

### Psychotherapy is hard to train and practice: too much conceptual information, too little embodied learning

*Truth is ever to be found in the simplicity, and not in the multiplicity and confusion of things.*

Sir Isaac Newton (1643–1727).

*A seeker asked the master, “Why does the path of healing grow more tangled and complex?”*

*The master replied, “Because the mind builds mazes to wander.”*

*“Then how shall we find simplicity?” the seeker questioned.*

*The master smiled gently, “Embrace curiosity.”*

*“But curiosity is not easy,” said the seeker.*

*“Sit in stillness,” the master whispered, “and listen to the quiet stirrings within.”*

ChatGPT (2025).

The development of a skillful and attuned therapist has long been recognized as a challenging endeavor. Since the beginning of the field, this training has required time-consuming efforts of transmitting concepts, frameworks, approaches, and demeanors that entail high degrees of complexity. Today, the demands have grown and the dimensions of knowledge required are continuously expanding.

In psychopathology, the increasing number of diagnoses and subdivisions demands that trainees and therapists familiarize themselves with an ever-increasing body of knowledge. Each new edition of the DSM or ICD has introduced additional diagnostic categories, and often along with them related controversies (Wakefield, 2016). For instance, the current DSM-5-TR (American Psychiatric Association, 2022) spans 947 pages and includes 541 diagnostic categories, a stark increase from the 106 categories in the DSM-I. Meanwhile, the whole categorical framework of psychopathology is increasingly challenged by the emergence of trans-diagnostic paradigms, which advocate for conceptualizing mental health through shared processes across disorders rather than classifications of distinct diseases (Dalgleish et al., 2020).

In psychodiagnostic and psychological assessment, there has been a steady proliferation of questionnaires, tools, and procedures. However, the most significant growth has occurred in treatment methods, theoretical models, intervention protocols, and therapeutic techniques—the core knowledge base for psychotherapists. Each accompanied by its own training requirements. Early advocates of the movement supporting evidence-based practice hoped that research would streamline these options, identifying the most effective treatments and simplifying the landscape. This vision incorporated the medical illness model, which assumes that latent disease entities have to be targeted with specific therapy protocols. Therefore, the aspiration to develop optimal, evidence-based protocols tailored to each of the DSM diagnoses, embracing a “one-protocol-fits-all (patients)” approach (Hofmann and Hayes, 2019). However, the reality has proven far more complex. By the mid-2010s, confidence in the ability to identify universally superior treatments was undermined by the consistently replicated findings of the “dodo bird effect” (Wampold and Imel, 2015). For instance, in the case of Major Depressive Disorder—one of the most prevalent mental health conditions globally—over 500 randomized controlled trials (RCTs) have shown that a wide variety of treatments, each based on purportedly distinct mechanisms, are equally effective, and as effective as drug therapies (Cuijpers et al., 2023). In response, a growing movement within the field has shifted toward a trans-diagnostic paradigm in psychopathology and a trans-theoretical approach to psychotherapy. This emerging perspective promotes the ideal of “a new therapy for each patient” (Norcross and Wampold, 2018) or “personalized/precision interventions” (Hayes and Hofmann, 2021; Moskow et al., 2023). It emphasizes moving away from treating psychiatric diagnoses as monomodal entities and toward addressing each patient’s unique biopsychosocial characteristics, goals and needs. Yet, a truly patient’s individual biopsychosocial approach would imply considering, at least to some extent, genetic and epigenetic factors as well as cultural and socioeconomic ones. Tellingly, the frequency of the use of the word “complexity” as a descriptor in current literature (the present paper included)—spanning for example from the cognitive/constructivist “4E” model (Newen et al., 2018) to the behavioral “process-oriented” model (Hofmann and Hayes, 2019)—underscores the magnitude of this paradigm shift.

As a result, psychotherapists and trainees face an overwhelming expansion of information, models, and an almost combinatorial explosion of treatment options. This breadth of material is difficult to cognitively process and practically manage, even for the most capable therapists and students. In addition, beside the sheer cognitive “load,” and in spite of the massive evidence-based research on the “dodo effect,” professionals and students often find themselves trapped into the biased polarisations resulting from the harsh competition, or even ideological conflict, between different clinical models, each proposing its own best practices. The obvious example being the decades-old counterposition of CBT vs. psychodynamic approaches, but more recently also the significant divergences within the same approach due to the proliferation of proposals.

## Embodied therapeutic relationship

In addition to the trend of growing conceptual complexity in psychotherapy, one of the most consistent findings in nearly a century of research is the central role of the therapeutic relationship, or alliance, in facilitating change. Decades of empirical evidence highlight the importance of the therapist-patient alliance for successful treatment, regardless of theoretical orientation (Safran et al., 2001; Wampold and Imel, 2015; Zilcha-Mano, 2017; Wampold and Flückiger, 2023). The correlation between the therapeutic relationship and treatment outcomes is among the most replicated findings in psychotherapy research—and perhaps in psychological science as a whole (Zilcha-Mano and Fisher, 2022; Flückiger et al., 2024). A recent large meta-analysis further confirms that a strong alliance is significantly associated with better outcomes, while a weakened alliance often leads to premature treatment termination (Del Re et al., 2021).

Importantly, emerging evidence suggests that the therapeutic relationship may not only correlate with, but also causally influence outcomes. This supports the notion of the alliance as a “change mechanism,” with the capacity to induce therapeutic change on its own (Zilcha-Mano and Muran, 2024). Acknowledging this, the APA Third Interdivisional Task Force on Evidence-Based Relationship and Responsiveness has drawn clear conclusions (Norcross and Wampold, 2018):

1. “The psychotherapy relationship makes substantial and consistent contributions to patient outcome independent of the specific type of psychological treatment.”
2. “The therapy relationship accounts for client improvement (or lack of improvement) as much as, and probably more than, the particular treatment method.”
3. “Practitioners are encouraged to make the creation and cultivation of the therapy relationship a primary aim of treatment, especially for relationship elements found to be demonstrably and probably effective.”
4. “Mental health training and continuing education programs are encouraged to provide competency-based training in the demonstrably and probably effective elements of the therapy relationship.”

The therapeutic relationship has been operationalized as encompassing constructs such: alliance, empathy, collaboration, positive regard, goal consensus, and fostering a real relationship, among others. An essential point has to be highlighted here: these constructs represent capacities that are personal as well as professional, abilities rooted in the therapist as a person. They are not tied to treatment methods, nor distinctive features of specific techniques or protocols (Hofmann et al., 2022).

Moreover, for these personal capacities to be effective, therapists must embody and enact them through their own body and mind. These capacities cannot be learned solely at a conceptual level as abstract principles or desirable attitudes. If treated as mere concepts, they risk becoming a checklist of abstract ideals or, worse, habits of pretended and simulated behaviors (Duquette et al., 2022). Pretending to be curious can be performative or worse, fall into the category of synchopathy—imitating genuine empathy and openness while lacking authenticity. Such behaviors, analogous to mannerism or ornamental affectation, often function as defensive mechanisms therapists use to mask insecurity, particularly impostor syndrome. In the competitive contemporary contexts of coaching, counseling, and psychotherapy, it can also be driven in part by financial incentives in the hope of securing a client. In both cases it undermines authentic curiosity, disrupts genuine therapeutic alliances, and ultimately compromises therapeutic effectiveness. Cultivating genuine curiosity through reflective, embodied practices remains crucial precisely because it helps therapists recognize and overcome these superficial and moralistically fabricated attitudes. Authenticity is not a condition reached once forever, it needs a moment to moment intentional effort in inquiring and bringing awareness to the motives and drives that are actually arising and passing through our body/minds, beyond our often complacent narratives.

At some point in our careers, we have all heard and tried (and even taught) the principle of “fake it, until you make it.” This may be suboptimal. Instead, these are advanced and refined interpersonal and intrapersonal competencies that require gradual cultivation. Developing them demands time, effort, patience, and an ongoing commitment to embodying them authentically. As Wampold and Imel (2015, p. 176) aptly stated, “The essence of psychotherapy is embodied in the therapist.”

## Personal practices

Developing effective therapeutic relationships, central for psychotherapists-in-training and practicing clinicians alike, implies several concurrent abilities: integrating conceptual knowledge, intuitive understanding, and clinical experience; cultivating and embodying at intrapersonal and interpersonal level those qualities operationalized in abstract constructs; personal integrity and authenticity; leveraging the healing potential of these qualities in practice. How do we balance all of these to harness their healing potential in practice?

One proposed strategy is the use of “personal practices,” defined as formal psychological interventions or techniques that therapists personally engage in experientially over an extended period, spanning weeks, months, or even years (Bennett-Levy, 2019). These practices can be done individually or in groups and focus on professionally oriented personal development. Research suggests that conventional training strategies, which emphasize cognitive learning and procedural application without incorporating personal practice, are insufficient for fostering these qualities. In addition to personal therapy, some of the most studied personal practices in the empirical literature include mindfulness meditation programs—encompassing practices such as mindfulness, mindful movements, compassion, and benevolence—and other self-practice/self-reflection programs (Bennett-Levy and Finlay-Jones, 2018).

## Balancing knowledge and relational skills

A key and ongoing tension in psychotherapy training and practice therefore lies in balancing the acquisition of conceptual knowledge—especially as the field continues to expand—with the development of skills necessary to build and sustain a therapeutic relationship. While significant time and resources are devoted to teaching theoretical frameworks and protocols, less attention is given to the intangible but critical factors underpinning connection, active listening, and relational depth.

It is especially important to strike this balance during training and early in one’s career. Factors that complicate the process include (a) insecurity in trainees at the start of training, due to a lack of knowledge (Bennett-Levy, 2019); (b) resistance to feedback, which may emerge during supervision or skill evaluation; and (c) intolerance of uncertainty, where trainees gravitate toward formulaic or “cookbook” approaches rather than embracing the inherent ambiguity and nonlinearity of therapy.

A common thread connecting these challenges is the therapist’s ego—a construct that can both hinder and facilitate therapeutic growth. For the purposes of this paper, ego can be operationally defined from a humanistic perspective as representing an individual’s (conscious and unconscious) self-concept, which shapes a sense of identity and how they perceive and respond to their internal experiences and relationships with others. In psychotherapy, when unseen, egoic factors can cloud judgment, interfere with deep listening, and encourage self-preoccupations, the self-focused need to be seen as competent and skillful that detract from focus on patient care (e.g., interruptions, overinterpretations, overexplanations). Research indicates that therapists may sometimes engage in discussions that deviate from the therapeutic focus, potentially impacting treatment effectiveness. For instance, a study evaluating community program therapists delivering motivational enhancement therapy (MET) found variability in adherence to MET principles, suggesting that some therapists might not consistently apply core techniques, possibly leading to conversations that are less relevant to therapy goals (Martino et al., 2008). Additionally, therapists have reported experiencing listening exhaustion when clients discuss topics that stray from the session’s focus. This suggests that managing and redirecting conversations to maintain therapeutic relevance is a recognized challenge in clinical practice (Ricks and Brannon, 2023). This can be especially challenging when running groups. Even among well-intentioned therapists, the ego can interfere with the therapeutic relationship or even unintentionally cause harm, partly due to the inherent power differential in the therapeutic relationship. If a therapist is acting on countertransference (conditioned reactivity) they may inadvertently engage in agonistic, manipulative, discriminatory, even abusive behaviors; or reinforce the client’s maladaptive cognitive distortions and beliefs.

Cultivating self-awareness becomes thus nothing less than a foundational and mandatory advanced professional competence. It helps trainees as well as seasoned therapists to recognize and address ego-based hindrances right in the moment. Self-awareness practices not only reduce defensiveness but also foster a healthy sense of self, curiosity, and openness—critical factors in establishing therapeutic rapport (Kottler and Carlson, 2014). This raises two important questions: What is the basis for these ego-based hindrances that get in the way of psychotherapy training and practice? How to practically cultivate self-awareness?

## What gets in the way of the therapeutic relationship? Predictive processing and confirmation bias: a new framework

From a neuroscientific perspective, the brain faces a fundamental challenge: the sensory information available at any moment vastly exceeds its processing capacity. Predictive processing theories, which have reshaped our understanding of brain function, propose that the brain addresses this issue by relying on top-down processing rather than exclusively interpreting bottom-up sensory input.

Historically, sensory information was thought to flow from the bottom up, with cortical structures organizing and interpreting signals. However, anatomical studies revealed that the brain has far more top-down than bottom-up connections. Predictive processing resolves this discrepancy by positing that the brain generates internal models of the world and uses these models to selectively gate sensory input. These generative models compare incoming sensory data to their predictions of what they expect (based on past experience), and they update only when a mismatch (prediction error) occurs. This process minimizes uncertainty while maintaining cognitive efficiency.

Generative models allow the brain to ignore most sensory information as irrelevant, relying on minimal input to (construct its world and) sustain its understanding of the world. For example, estimates suggest that more than 80% of our experience in any one moment is shaped by internal models rather than raw sensory data (Friston, 2010). In essence, our experience of a situation from moment to moment is constructed by our generative models. While this efficiency is advantageous, it also creates vulnerabilities, particularly in interpersonal contexts.

## Predictive processing in action: implications for psychotherapy training

Consider a workplace example: Jack and Jill are co-workers preparing for an important presentation. When Jack asks Jill a question, she gives a brief answer and moves on. Depending on Jack’s internal model of their relationship, he may interpret this interaction in different ways. If their working relationship is strong and characterized by trust, Jack might perceive Jill’s brevity as efficient and aligned with their shared goal. Conversely, if Jack doubts Jill’s regard for him, he might interpret her response as dismissive or irritated, potentially leading to conflict.

This example highlights the double-edged sword of predictive processing: while generative models enhance efficiency by filtering sensory input, they also predispose individuals to error based on cognitive biases, especially in ambiguous situations. Long before predictive processing was articulated conceptually, and before neuroscience was even a field of study, Peter Wason identified and termed this type of interpretation “confirmation bias” (Wason, 1960). Confirmation bias is a cognitive bias that refers to the tendency of people to favor, search for, interpret, and remember information in a way that confirms their pre-existing beliefs or hypotheses. Jack’s generative model—in service of efficiency—is more likely to bias incoming sensory information (short answer) in a way that confirms his beliefs of Jill (she’s annoyed, etc.).

In psychotherapy, predictive processing can influence both therapists and patients. Trainees may default to rigid, formulaic approaches when faced with uncertainty, relying on their generative models to interpret ambiguous patient behaviors or feedback. Worse, “seasoned” therapists who are not aware of this process are at even more risk of solidification of their views, identities, and ease of doing things “a certain way” when faced with the double-edged sword of expertise. This cognitive rigidity can undermine therapeutic relationships by reinforcing biases, interpreting unfolding experience through simplistic schemas, and perpetuating ineffective and potentially detrimental habits.

Moreover, predictive processing theories shed light on the fact that resistance to change is a deeply human trait and can manifest strongly in psychotherapy training (Di Bartolomeo et al., 2024; Villiger, 2025). Updating deeply ingrained mental models (both cognitive and metacognitive) requires significant cognitive effort and can disrupt psychological equilibrium, leading to discomfort. Emotional attachments to beliefs about oneself or the therapeutic process can further reinforce resistance, as these beliefs provide a sense of identity and security (Jackson and Jones, 2025).

Helping trainees and therapists to recognize and contextualize predictive processing and related biases—such as confirmation bias—can enhance their ability to self-reflect and adapt. By understanding how their minds and brains process and filter information, trainees can cultivate greater openness and flexibility, improving both their clinical practice and their ability to form meaningful therapeutic relationships. Supervisors and curriculum developers can use these insights to foster a training environment that emphasizes curiosity, self-awareness, and adaptability over rigid adherence to protocols. Mindfulness training can be particularly well-suited for these types of training environments.

## Rigidity is a two-way street: the essential role of developing flexibility for both therapist and patient

Many individuals suffering from mental health challenges share a core difficulty: deeply ingrained emotional and relational patterns, often charged with painful emotions, that are resistant to change. Rigidity has been defined as “the tendency to develop and perseverate in particular emotional, cognitive, and behavioral patterns, employing them continuously in situations where the pattern is no longer effective” (Morris and Mansell, 2018, p. 3). This rigidity reflects both a tendency to repeat cognitive, emotional and behavioral maladaptive processes across contexts and a loss of intentional control over these patterns, rendering them increasingly automatic.

This phenomenon has been recognized since the early days of clinical psychology by the founding fathers. Sigmund Freud introduced the concept of “repetition compulsion” (*Wiederholungszwang*), describing an unconscious drive to repeatedly enact distressing thoughts and behaviors that were difficult or distressing earlier in life. Similarly, Pierre Janet explored the relationship between awareness and different levels of automatic mental processes in several pathological phenomena, including “dissociation,” a term coined in his 1889 seminal study L’Automatisme Psychologique (Ellenberger, 1970).

More recently, research has converged on the idea that diverse forms of psychopathology share core features and underlying factors that maintain emotional suffering. Genetic, neurophysiological, and personality studies have supported this transdiagnostic perspective (Buckholtz and Meyer-Lindenberg, 2012). Transdiagnostic processes—cognitive, emotional, and behavioral mechanisms that contribute to distress across disorders—have been identified as central to psychological suffering (Nolen-Hoeksema and Watkins, 2011). However, evidence suggests that these processes do not inherently cause distress or disability. Instead, it is the rigidity of their expression—how inflexibly they are applied—that determines their pathological impact (Morris and Mansell, 2018; Schultz and Searleman, 2002; Bickhard, 1989). This insight from research implies that reducing rigidity by developing flexibility can mitigate the negative effects of these processes and promote psychological well-being (Giommi et al., 2023; Westhoff et al., 2024; Kashdan and Rottenberg, 2010). However, rigidity is a two-way street: it is essential to develop flexibility for both therapists and patients. Given the role of the therapeutic relationship, a rigid therapist’s mind cannot foster flexibility in a patient’s mind.

## The default response to complexity: more complexity

So far in the field of psychotherapy the default response to the challenge of increasing complexity has mostly consisted in attempting to elaborate more sophisticated, articulated, apt concepts. That is, the attempt to manage complexity often results in introducing even more complexity. Just as an example of this general tendency, we quote a few lines excerpted from a recent paper: “This coherent approach to more transtheoretical and integrative concepts of clinical training and practice provides a firm foundation by targeting biopsychosocial processes of change, analyzing these processes using an idiographic complex network approach, and organizing findings on the intellectual agora of multi-dimensional and multi-level evolutionary science” (Hofmann and Hayes, 2024).

While this complexity-based approach is effective in many scientific fields guided by a “third-person” epistemological framework, it becomes less appropriate—and potentially counterproductive—when applied to the exploration and understanding of inner life. In areas like psychotherapy, where “first-person” and “second-person” perspectives are also essential, relying solely on this default approach may be insufficient (and it might betray unrecognized epistemological and ideological assumptions carried over by the positivistic promise of objectivity etc.) (Varela et al., 2017; Shear and Varela, 1999).

Doubts about the viability of this “more of the same” (in the sense of more complexity) strategy are not new. At the dawn of scientific psychology, William James, another founding figure in psychology, warned against this tendency to over-rely only on concepts. In 1909 he observed, “The essence of life is its continuously changing character; but our concepts are all discontinuous and fixed, and the only mode of making them coincide with life is by arbitrarily supposing positions of arrest therein. With such arrests our concepts may be made congruent. But these concepts are not parts of reality, not real positions taken by it, but suppositions, rather notes taken by ourselves, and you can no more dip up the substance of reality with them than you can dip up water with a net, however finely meshed” (James, 1977).

Instead of layering more concepts, James advocated for simplicity: grounding oneself in the lived reality of “passing moments” rather than in intellectual constructs.

## Understanding rigidity for cultivating flexibility: open/expanded vs. closed/contracted

To address rigidity, it is essential to understand its origins. As seen, the mind’s default tendency is to “go with what we know,” adhering to established generative models of the world. While this strategy promotes cognitive efficiency, unchecked rigidity can lead to maladaptive patterns that span the range from a tendency to inflexible habits to manifest psychopathology.

Before addressing the causes and conditions underlying rigidity, therapists and patients must first become familiar with its mental and physical manifestations—as well as those of its opposite, flexibility. As articulated in Antico et al., prior research suggests that openness versus closedness can serve as a simple, descriptive, embodied, and, notably, in-the-moment marker of these states (Antico et al., submitted). When asked, “Do you feel open/expanded or closed/contracted right now?” individuals can often non-conceptually and intuitively recognize their experience as aligning with one category more than the other. For example, when queried via questionnaire if one had to pick if anxiety feels more in the category of open/expanded vs. closed/contracted, our previous research shows that without operationally defining these categories, individuals rate it with an average of 6.9/10 as closed/contracted (*N* = 303). As a contrast, curiosity was reported with an average of 7.7/10 as open/expanded (*N* = 310); kindness was reported with an average of 8.4/10 as open/expanded (*N* = 337) (Antico et al., submitted). This qualitative method—which can be viewed almost as a therapeutic “micro-intervention”—invites individuals to explore their direct experience instead of falling into the potential trap of an exceedingly conceptual understanding or judgment of their experience.

For therapists, cultivating an awareness of openness and closedness in themselves can be a powerful tool. By recognizing moments when they feel “closed down” or feel a direct sense of contraction in a session, therapists can identify how rigidly they are holding onto a particular idea, concept, or story. In the context of predictive processing, this may reflect an attempt to force sensory input to align with an entrenched generative model.

Additionally, therapists can train themselves phenomenologically to notice their own state shifts (e.g., sudden transitions from openness and flexibility to closedness and contraction). Such awareness constitutes a phenomenological reduction, temporarily suspending the habitual reification of cognitive and emotional patterns (i.e., their stories or mental models of the world) as objective realities, and instead observing them directly as lived experiences. These experiential shifts serve as markers indicating therapists’ blind spots or countertransference reactions, offering valuable insights into the therapeutic process. Similarly, therapists can invite patients to cultivate this phenomenological awareness, guiding them to recognize when they are falling into rigid, contracted patterns. In doing so, clients become better able to experience anxiety and related states as dynamic, embodied phenomena rather than fixed cognitive or somatic realities, creating meaningful opportunities for intervention, insight, and change.

In many aspects deeply resonant way to relate with the actuality of our experience can be found in the richness of the phenomenological tradition. Which invites us to “reduce,” or suspend, our “natural attitude,” the automatic tendency to reify our cognitive patterns and expectations, and to explore what emerges in this “suspension.” Contemporary phenomenological approaches are precious to the extent they have developed operative methods to train apprentices to enact these “inner gestures.” These methods, like neurophenomenology and micro-phenomenology, are altogether awareness practices (see for example Poletti et al., 2024; Petitmengin et al., 2019; Bitbol and Petitmengin, 2017). Indeed, these examples can be accurately described as phenomenological, as they involve therapists and clients learning to momentarily suspend their habitual attitudes and recognize cognitive patterns as experiences rather than objective realities. The process described—shifting awareness from being caught in rigid patterns to observing these patterns as experiential phenomena—aligns closely with phenomenological reduction. Such a reduction facilitates the recognition of anxiety not merely as a fixed state, but as a dynamic, embodied experience alternating between openness and contraction.

## As simple as curiosity (?)

In the present paper we are proposing that instead of dealing with complexity by introducing more complexity, an alternative path, at the same time practical, dynamic and lively, can be discovered by moving in the opposite direction: simplicity.

William James, again, can inspire us: “If what we care most about be the synoptic treatment of phenomena, the vision of the far and the gathering of the scattered like, we must follow the conceptual method. But if, as contemplative, we are more *curious* about the inner nature of reality or about what really makes it go, we must turn our backs upon our winged concepts altogether, and bury ourselves in the thickness of those passing moments over the surface of which they fly, and on particular points of which they occasionally rest and perch” (*A Pluralistic Universe*, 1977; italics ours) (Table 1).

**Table 1:** Comparison of existing theoretical concepts to the proposed framework.

Existing concept	Explanation	This paper’s addition
Therapeutic alliance as predictor (Safran et al., 2001)	Alliance quality predicts therapy outcomes and rupture repair interventions address disruptions.	Curiosity as a modifiable mediator that dynamically enhances alliance quality throughout therapy.
Insight as outcome (traditional models of therapy)	Insight emerges as a final therapeutic goal or result of accumulated interventions.	Insight as an active, embodied process continuously arising through curiosity-driven interactions during sessions.
Genuineness as stable therapist trait (Rogers, 2012)	Therapist genuineness is considered a consistent and authentic personality characteristic.	Genuine curiosity as a trainable, experiential skill therapists can deliberately cultivate and strengthen.
Alliance repair as rupture-intervention technique (Safran and Muran)	Techniques specifically designed to address and resolve relationship disruptions when they occur.	Curiosity as a preventive and ongoing relational stance, proactively fostering openness and resilience, thereby reducing ruptures.
Empathy and acceptance as therapist stance (Rogers)	Empathy and unconditional acceptance form the relational foundation therapists provide to clients.	Curiosity as intentional stance enhancing empathy, acceptance, and relational depth through active exploration of client experiences.

In psychotherapy a direct and practical way to pursue simplicity is through curiosity. In contrast to complex concepts, curiosity provides a direct, simple path to engage with experience. Curiosity is a natural human quality—readily available, intuitive, and infectious. It does not require extensive learning and intellectual effort. When psychotherapists learn to trust authentic, caring curiosity, they naturally embrace simplicity. Yet, the simplicity of curiosity is not easy. Genuine curiosity can run counter to deeply ingrained habits–which by nature are rigid and automatic–and thus can require consistent cultivation until it becomes the habit itself. Although always potentially available to us, curiosity must be embodied and enacted, not merely understood intellectually. Curiosity requires active engagement and *practice.*

Curiosity can also be scientifically understood through two complementary types: deprivation curiosity and interest curiosity (Litman, 2007). Deprivation curiosity arises from a need to fill an informational gap, often driven by an uncomfortable sense of “not knowing.” This form of curiosity is like an itch relieved when scratched and is associated with dopaminergic reward pathways. In contrast, interest curiosity is fueled by the joy of exploration and learning. It is less urgent and more open, akin to enjoying the journey rather than focusing solely on the destination. Interest curiosity aligns more closely with fostering flexibility and agility, as it encourages therapists and clients alike to remain present and engaged in the process rather than fixating on specific outcomes (Whitecross and Smithson, 2023; Kashdan and Rottenberg, 2010).

## Cultivating curiosity in psychotherapist training and practice

Therapists often encounter patients who are desperate to understand their suffering, driven by the question, *Why am I feeling this way?* This need for explanation can dominate therapy, leading to a focus on piecing together facts from a client’s past and present. While this approach can feel productive, it often leads to a dead end. Like using ice cream to soothe anxiety, gathering facts and insisting to make sense through explanations may temporarily satisfy but rarely addresses the root causes of suffering (Brewer, 2024).

Curiosity offers a different path. Instead of amassing facts, therapists can model and cultivate curiosity in themselves and their clients. A meta-analysis by Schutte and Malouff (2023) reviewed 41 randomized controlled trials and found that interventions designed to enhance curiosity had a significant positive effect (Hedges’ g = 0.57) on increasing individuals’ levels of curiosity.

This begins in training. For example, when a trainee feels stuck—whether in making a diagnosis or developing a treatment plan—a supervisor can model curiosity by exploring the trainee’s present-moment experience. Rather than focusing on “getting it right,” the supervisor can encourage awareness of unfolding thoughts, emotions, and bodily sensations, fostering openness to ambiguity. This approach teaches trainees to value the process over the outcome, strengthening their ability to be present with clients.

Curiosity also has a contagious quality. When supervisors embody genuine curiosity, it spreads to trainees, and eventually to clients (Gorle, 2023; Kramer et al., 2014). For example, instead of providing solutions or interpretations, a therapist can ask exploratory questions that invite clients to connect with their immediate experience. This not only reduces rigidity by challenging entrenched generative models but also empowers clients to trust their own inner wisdom. Importantly, the development of inner wisdom is a dynamic and iterative process, in which cognitive biases are increasingly identified and let go of during therapy. This is in contrast to “false” wisdom or insight that comes from and perpetuates cognitive biases.

## Curiosity, the cure for rigidity (there is no cure for curiosity)

*“THE CURE FOR BOREDOM IS CURIOSITY. THERE IS NO CURE FOR CURIOSITY” –Ellen Parr (Reader’s Digest, 1980).*

Curiosity naturally promotes openness and is a marker of flexibility (Silvia and Christensen, 2020). Therapists can therefore use the tool of open/closed experiential framework to gauge their own flexibility and that of their clients. Genuine curiosity signals openness, while rigidity often coincides with a lack of curiosity. Importantly, fostering curiosity requires confidence in “not knowing”—a quality that can be particularly challenging for trainees who feel pressured to provide answers and appear competent.

Additionally, by embracing curiosity, albeit counterintuitive to a novice’s expectations, when therapists embrace curiosity, they shift from a position of authority to one of cooperation, empowering clients to explore their experiences and discover their inner resources. This process helps both parties step out of rigid generative models, reducing confirmation bias and fostering shared moments of discovery and insight. The journey is the destination. Curiosity, when cultivated with sincerity and care, transforms therapy into a shared exploration—one that is both simple and potentially profound.

## Interoceptive awareness as a professional tool

If curiosity is such a critical skill, how can it be fostered? An effective way—beyond intellectual or cognitive processes—is by cultivating interoceptive awareness. This can be seen as a generalization extending the open/closed experiential framework. It involves learning to trust the body as a rich source of information and guidance, and moving beyond an exclusively mental or intellectual approach to psychotherapy. By paying attention to bodily sensations, therapists can be guided by a felt sense rather than over-relying on conceptual frameworks. For instance, with training, the body serves as an accurate instrument to check if one is in “open/expanded” or “closed/contracted” mental states. Feeling closed or contracted always correlates with sensations of tightness, tension—literal markers of contraction—or restlessness. In contrast, open or expanded states are often associated with sensations of ease, lightness, or spaciousness, which can feel like a gentle relaxation of tension or a sense of energy flowing freely (Antico et al., submitted). The latter can also be markers of interest curiosity.

Many people are unaware of the rich, pre-semantic, non-intellectualized information continuously offered by their bodies. Cultivating interoceptive awareness helps therapists recognize these signals, fostering a deeper connection to themselves, their clients, and the therapeutic process. Expanding interoceptive awareness is a foundational skill for psychotherapists across theoretical orientations (both for themselves, and their patients). A body of recent research has highlighted the importance of bodily awareness in improving emotional regulation and facilitating change (Nord and Garfinkel, 2022; Khalsa et al., 2018). Interoceptive awareness can inform the therapeutic process in many ways; notably it informs and is central to many therapeutic “bottom-up” modalities (e.g., Internal Family Systems, Somatic Experiencing, Focusing, etc.). Mindfulness meditation and mindfulness-based programs (MBPs) have specifically emphasized practices that develop proprioceptive and interoceptive awareness (Price and Weng, 2021; Farb et al., 2015). For the purpose of simplicity, in this paper, we focus on how interoceptive awareness interacts with and is fostered by curiosity.

## Practical applications of interoceptive awareness to foster curiosity in psychotherapy

It is possible to cultivate curiosity without the need of a formal mindfulness practice.

For example, a practical way to refine curiosity through interoception is to learn to pay attention not only to the patient but also to one’s own bodily experience during interactions. This can help therapists discern whether their inquiry is grounded in genuine (interest) curiosity, or driven by other motivations such as a need for a sense of control or a cognitive/analytical understanding. Two practical guideposts for checking authentic curiosity are:

1. Monitoring for sensations of contraction/closedness versus expansion/openness.
2. Simply noticing if the therapist’s questions are driven by deprivation curiosity (e.g., filling an informational gap), narrowing the discussion, or seeking to fulfill their own need to understand.

By leaning into interoceptive cues and temporarily stepping out of intellectualization during the practice of interoceptive awareness, therapists can better navigate the unfolding therapeutic process. Over time, this practice helps therapists trust the journey of not-knowing, allowing clarity to emerge organically, not just “extracted.” Cultivating patience—and recognizing impatience as a form of contraction—is also a key skill in this process.

## An exercise in interoceptive awareness to cultivate curiosity

The following dyadic exercise, as administered during psychotherapy or other types of training, can help therapists to learn how to trust interoceptive awareness and cultivate authentic curiosity:

1. *Speaker*: Recall and describe a recent emotionally charged situation with continued personal significance (positive or negative).
2. *Listener*: Sit in silence, just in front of your partner, listening with open and relaxed attention. While listening to the other, at the same time as best as you can continuously monitor your own body for interoceptive signals, until you feel (or not) the bodily sensations arising and resonating with a sense of genuine curiosity (e.g., sensations of opening or leaning in). Wait for curiosity to arise naturally, before asking a question generated from it. Ensure that questions come from this impulse of genuine interest rather than an effort to lead or control the conversation. No need to force curiosity. If no questions arise, just continue listening. As a note, a common impediment for the listener is being stuck in their head instead of resting in awareness in their body (e.g., analyzing).
3. Continue the exercise for 5–7 min, then pause a minute to take mental notes of what you both have observed in the process, and reverse roles.
4. Finally, share what you have noticed and reflect together on:

- The effects of being listened to in this way.
- The effect of the questions asked (if any) as emerged in this way.
- The experience of being the listener and trusting interoceptive signals.
- The challenges or insights that arose in trusting authentic curiosity to guide questions.

## Curiosity is simple, but not easy. The support of a formal practice

While curiosity is innate at birth, it often decreases while growing older as societal expectations and roles and habitual patterns push toward rigidity. Genuine curiosity in adulthood requires refined self-awareness and the confidence to embrace not-knowing. To counterbalance this unavoidable tendency toward rigidity, a systematic and enduring practice—like mindfulness meditation—can be very useful, or indeed needed, to rekindle curiosity and foster a more flexible, open mindset.

## Openly receptive presence (mindfulness) as authentic curiosity

Mindfulness, defined as “an openly receptive presence that enables a full taking in” of present moment experience (Anālayo, 2020a,b) is inherently simple and naturally inquiring.

Mindfulness meditation offers a systematic and scientifically validated method to cultivate attentive curiosity and penetrative awareness. It is not merely about monitoring the present moment; it is a form of knowing that fosters insight. Importantly, while mindfulness can be associated with spiritual practices, even secular and introductory programs like MBSR have demonstrated the evidence-based potential to reduce suffering, loosening the grip of habitual reactions through insight (De Vibe et al., 2012).

This deconditioning potential begins with the simple act of paying attention to experience as it unfolds.

In Buddhist psychology, namely in the “early Buddhist teachings,” *vipassanā*, or “liberating insight”—is a penetrative understanding of the nature of experience that transcends mere, usual conceptual knowledge. Insight or intuitive comprehension is the key factor for deconditioning our mind. “This insight is not knowledge in the general sense, but penetrative knowledge acquired as a result of not looking *at* but looking *through* things” (Anālayo, 2012, p. 83). In other words, rather than accumulating facts or constructing intellectual interpretations, this form of knowing arises from directly witnessing and monitoring phenomena as they unfold, revealing deeper patterns and truths that cannot be grasped solely through analytical thought. Over time, sustained mindfulness (*sati*) is associated with intuitive and clear comprehension (*sampajañña*), which allows practitioners to see their experience clearly within its broader context (Anālayo, 2019; Anālayo, 2020a,b). “A clear and intuitive comprehension or apprehension of whatever is manifesting in the present experience, moment by moment. Even the clear apprehension in the moment of the presence of confusion, uncertainty, insecurity in our mind: recognizing these states are *like this now*” (Sumedho, 2004). Intuition or insight has long been acknowledged as a radically distinct mode of knowing in Western philosophical and scientific traditions as well (Giommi and Barendregt, 2014; Kounios and Beeman, 2014). Pragmatically, mindfulness is one of the most scientifically validated of the various methods currently available for training.

We can be inspired and supported by the ancient wisdom, particularly as transmitted through the early Buddhist traditions and its contemporary expressions (Anālayo, 2022a,b), which offers an articulated, precise and penetrating map of the mental qualities inherently present in sustained mindfulness (sati), that the practice gradually discloses. The cultivation of mindfulness naturally gives rise to seven “factors” meant as wholesome, liberating qualities inherent in awareness and fostering insight. These qualities emerge when the mind becomes less conditioned and more free (i.e., less rigidly driven by top-down predictive processing), providing a natural framework for meditative yet also for therapeutic development (Anālayo, 2017; Dhammadinnā, 2014). Of particular relevance to psychotherapy, in our view, is *dhamma-vicaya,* the “investigation of phenomena.” This factor – which has at times been translated by Buddhist scholars as curiosity (Akincano, 2019) – is a penetrative and sustained inquiry into experience, guided by “wise attention.” It describes a quality of mind characterized by openness and genuine interest. This ancient wisdom “map” suggests that if this quality is innate and spontaneously arising in sustained mindfulness, then it may as well be present and manifesting, at least to some degree, in authentic curiosity, and thus be available to anyone who investigates experience with this kind of curiosity. Curiosity, to be clear, does not require a formal meditative practice, yet can be inspired, informed and enhanced by meditative practices. Importantly, this exploration arises from compelling and fresh curiosity about the nature of things, particularly suffering, rather than a desire to “solve” immediate problems (Brewer et al., 2013). When therapists embody these qualities of awareness and investigation, they create the conditions for curiosity and insight to arise naturally, both in themselves and their clients (see Brewer et al., 2013, for a proposed explanation of specific mechanisms of how this works).

## Insight as process and outcome

Curiosity and insight are intimately linked. Insight is often understood as a specific realization (e.g., uncovering the root of a pattern), but it can also emerge as an ongoing process of discovery. In therapy, this distinction is critical. Instead of aiming for a predetermined destination, curiosity opens the therapeutic space for co-creation, empowering clients to explore their own experience and uncover their inner wisdom (as noted before, the development of wisdom is focused on the process and not the accumulation of content/knowledge). This process of mutual discovery transforms the therapeutic relationship, fostering empowerment, trust, and meaningful change. By prioritizing curiosity and intuitive awareness, therapists can move beyond rigid models and techniques, allowing the therapeutic journey itself to become a source of healing and insight.

## Psychotherapy as a performing art, not a routine

Psychotherapy cannot rely solely on concepts, protocols, manualized procedures, and techniques; it is not a routine or a “cookbook” approach. Much like generative models, which compel repetitive actions, this rigidity stifles creativity and responsiveness in therapy. Instead, psychotherapy might be better and more usefully understood as a performing art.

Consider the analogy of piano performance. A potentially gifted piano student needs several conditions to reach their potential: deep motivation, access to quality education and mentorship, dedicated practice, and exposure to inspirational models, such as masterclasses with accomplished performers. Yet, even with all these elements in place, one essential factor remains: “presence/mindfulness.” Each time, again and again, the performer steps onto the stage, their ability to access a condition of presence/awareness, the quality of the state of consciousness, deeply influences whether the performance transcends technical precision to become truly expressive. With poor presence, even a flawless performance risks feeling lifeless and routine. “Everything is written in the score, except the essential” wrote Gustav Mahler, the great Austrian composer.

Likewise in psychotherapy: “everything is written in the protocol, except the essential.” The quality of a therapists’ self-awareness and presence is the enabling condition for fully embodying and expressing their potential, their skills and knowledge. It is the cornerstone of therapeutic effectiveness (Giommi, 2024).

## The goal of therapy: obsolescence by design

An ideal outcome of therapy is its eventual obsolescence. Markers of successful therapy traditionally include symptom reduction (e.g., anxiety or depression), therapeutic insights, and signs of internalization, such as the patient anticipating what the therapist might say or reporting an internalized version of the therapist’s guidance (e.g., “I could hear your voice in my head”). However, as seen, internalization carries risks when driven by the therapist’s ego (e.g., the patient overidentifying with the therapist’s wisdom) or the patient’s insecurity (e.g., dependency on the therapist in the short term and long term). As such, self-awareness (and self-esteem) is critical for the therapist, such that they can clearly identify, and not rationalize or distort their own motivations. Critical motivations to monitor include financial incentives, preference for “easy” patients who minimize uncertainty, seeking admiration or affirmation from patients, potential pull toward exerting power or control, avoidance of potential conflict, people-pleasing, and sympathizing with patients who have the same types of views (i.e., therapizing in an echo chamber).

Curiosity acts as a natural antidote to these ego-driven tendencies. At the same time, curiosity naturally builds a more solid basis of operation; as highlighted earlier, curiosity not only feels good, but because the therapist never needs to know the answer, provide a pithy interpretation, or even be seen as a font of knowledge or bastion of wisdom, it reduces the pressure to be right, wise or even anyone at all. By transiently removing the need for ego-based interactions, curiosity reduces the therapist’s compulsion to provide answers, interpretations, or displays of wisdom. Instead, it focuses energy on cultivating the patient’s own wisdom and resources, reducing dependence on the therapist while fostering autonomy.

Industrial designed obsolescence in appliances refers to the practice of intentionally designing products with a limited lifespan, so they become outdated, break down, or require replacement after a certain period. This strategy encourages consumers to purchase new appliances more frequently, benefiting manufacturers but often leading to increased waste and environmental impact.

The concept of designed obsolescence can be subverted for psychotherapeutic benefit: instead of intentionally (or in most cases, unconsciously) designing therapy to become codependent or in continuous need of a “tune up,” if built in from the ground up, curiosity carefully crafts a vehicle designed for the development of a patient’s own wisdom. With the manual of how one’s own mind works and equipped with tools of internalized curiosity, self-repair, tune-ups, and augmentation become autonomous.

As such, over time, the therapist-patient relationship flattens from hierarchical (therapist holding the knowledge, wisdom and power) to peer-peer: fueled by curiosity, the therapist models and “trains” her patient to self-drive the vehicle of lifelong discovery.

## Discussion

**Figure 1:**
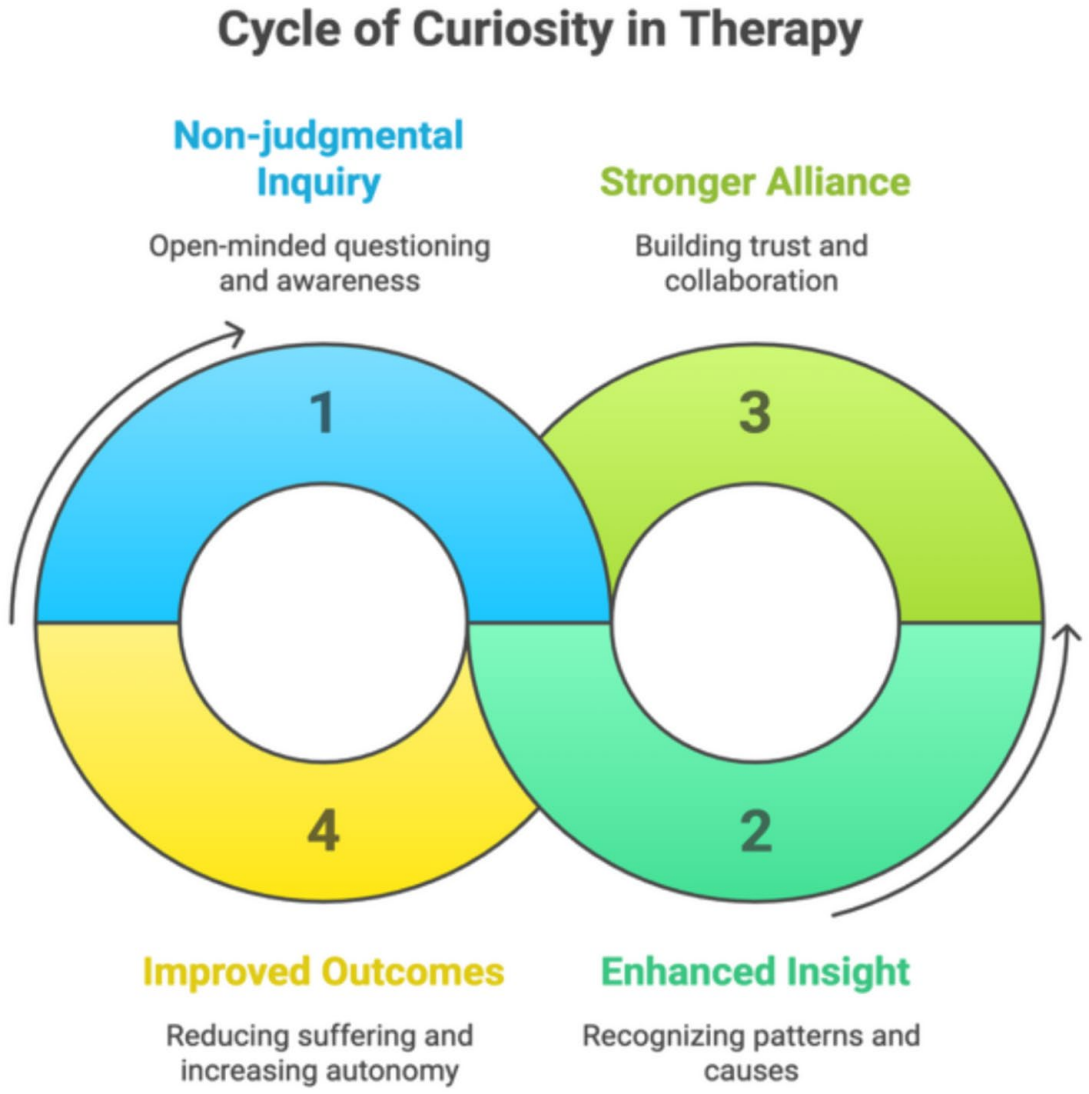
Cycle of curiosity in therapy.

In this paper, we articulated pitfalls of growing complexity in psychotherapy training and professional practice, and suggested a paradoxical approach that is simple and inherent in skillful interaction: curiosity. Theoretically, from a mechanistic perspective, curiosity directly targets key predictive processing elements that prevent therapeutic change such as confirmation bias. Pragmatically, emphasizing and incorporating curiosity into psychotherapy training may simplify treatment approaches, increase therapeutic alliance, and increase interoceptive awareness in therapists and patients alike. These elements may support and feed further deepening cycles of curiosity, insight and therapeutic alliance (see Figure 1).

This figure illustrates a dynamic, self-reinforcing process through which curiosity contributes to therapeutic change. Beginning with non-judgmental inquiry, curiosity promotes open-minded questioning and embodied awareness. This fosters enhanced insight by helping both therapist and client recognize patterns and causes of suffering. Insight, in turn, strengthens the therapeutic alliance by deepening trust and collaboration. A stronger alliance supports improved outcomes—reducing suffering and increasing autonomy—which then reinforces the therapist’s and client’s capacity for further curiosity. From a mechanistic standpoint, curiosity targets key elements of predictive processing, including confirmation bias and cognitive rigidity. When explicitly cultivated in therapy and training, curiosity may simplify interventions, deepen interoceptive awareness, and promote a spiraling cycle of growth and relational depth.

Curiosity may transform the therapeutic relationship by fostering shared discovery, empowering patients to trust their inner wisdom, and reducing dependency on external validation. When cultivated, curiosity becomes the foundation for both therapist and patient to navigate the therapeutic process with openness, presence, and resilience, to the benefit of both–and expanding to others by their unfolding and continually expanding way of being.

We are not suggesting that interest curiosity alone is sufficient to train effective psychotherapists. Traditional competencies—such as understanding theoretical frameworks, psychopathology, therapeutic models, and techniques—remain essential. Additionally, while curiosity can help a therapist stay calm in intense situations, so that they can keep cognition at full capacity, there may be situations and conditions where it is insufficient. For example, in cases such as severe personality disorders when a structured protocol is needed, the protocol can be followed with a stance of curiosity instead of mechanistically “following protocol.”

However, we suggest that while these conditions may be necessary, they are not sufficient for successful outcomes—defined not merely as a reduction in symptoms, but as a transformed relationship with them, grounded in insight, flexibility, and resilience. Within the therapeutic relationship, the therapist’s ability to begin with and sustain mindful curiosity plays a crucial role in facilitating change.

Genuine curiosity fosters a range of beneficial components that converge to enable insight. Insight transcends cognitive understanding, offering an intuitive, embodied sense of the causes and conditions that not only lead to but also maintain suffering. It also illuminates the possibility of freeing oneself from maladaptive patterns and experiencing ease in new ways (Brewer, 2019, 2024; Bauer et al., 2025; Brewer et al., 2013). In Buddhist psychology, mindful curiosity is similarly powerful because it generates a suite of mental qualities essential to reduce ego-driven obstructions to the mind’s liberative potential (Berkovich-Ohana et al., 2024; Gallagher et al., 2024).

While curiosity may seem simple, it is not easy. Curiosity requires ongoing cultivation through sustained practice to counteract the default tendencies of rigid habits and predictive processing. Rather than relying on pre-set question types like “What if?,” our view of curiosity centers on *being with* the client in a way that allows authentic, emergent inquiry to arise from the immediacy of their experience. This form of curiosity is not about generating clever or strategic questions, but about attuning to what is unfolding in the moment and allowing questions to surface naturally out of genuine interest and not-knowing. Curiosity, in this sense, is less a toolkit of prompts and more a trainable capacity for presence—cultivated through practices like mindfulness, embodied awareness, and supervision that highlights felt shifts in the therapeutic encounter. These inner conditions make space for curiosity to guide therapists toward deeper understanding, rather than steering the session toward preconceived goals.

Programs that train individuals in mindfulness—such as Mindfulness-Based Stress Reduction (MBSR), other mindfulness-based interventions (MBIs), and relational mindfulness practices like Insight Dialogue—offer structured, evidence-based approaches to cultivating the presence (Surrey, 2016) and self-awareness necessary for genuine therapeutic curiosity. Yet these are not the only pathways. Approaches rooted in phenomenological and experiential traditions, including Gendlin’s Focusing, Somatic Experiencing (Levine), Internal Family Systems (Schwartz), the Hakomi Method (Kurtz), and Person-Centered Therapy (Rogers), similarly emphasize the suspension of habitual judgments and the cultivation of direct, embodied inquiry. At their core, these methods align with Husserl’s concept of *epochè*, which invites practitioners to set aside preconceptions in order to perceive experience freshly (Husserl, 2012; Original work published 1931). Whether through mindfulness or phenomenological training, what matters most is the therapist’s commitment to exploring curiosity as a relational, felt, and trainable capacity—one that not only deepens clinical insight, but may also make the process a little more enjoyable for all involved.

## Data Availability

The original contributions presented in the study are included in the article/supplementary material, further inquiries can be directed to the corresponding author.
